# Supplementary material for: Estimation of district-level fertility using age-structured census data and assessment of spatial–socioeconomic differentials in Botswana, 2022
Source: PLoS One. 2026 Feb 5;21(2):e0342090. doi: 10.1371/journal.pone.0342090 (PMC12875440; doi:10.1371/journal.pone.0342090)
Supplement: Attachment — Submitted filename: PONE-D-25-55917-AF.pdf [file pone.0342090.s001.pdf]

PLOS ONE

Estimation of District-Level Fertility using Age-Structured Census Data and Assessment of Spatial–Socioeconomic Differentials in Botswana, 2022

--Manuscript Draft--

**Manuscript Number:**	PONE-D-25-55917
**Article Type:**	Research Article
**Full Title:**	Estimation of District-Level Fertility using Age-Structured Census Data and Assessment of Spatial–Socioeconomic Differentials in Botswana, 2022
**Short Title:**	District-Level Fertility Estimation and Spatial–Socioeconomic Differentials in Botswana, 2022
**Corresponding Author:**	Tiro Theodore Monamo, MA<br>University of Botswana<br>Gaborone, BOTSWANA
**Keywords:**	Fertility estimation<br><br>Age-structured census data<br><br>Spatial differentials<br><br>Socioeconomic inequalities<br><br>Botswana
**Abstract:**	Background<br><br>Reliable subnational fertility estimates are essential for guiding demographic policy and planning. In Botswana, national fertility decline has been well documented, but less is known about district-level variation and the socioeconomic factors underpinning it. This study pursued three objectives: (i) to estimate district-level fertility from age-structured census data, (ii) to validate these estimates against established indirect or model-based techniques, and (iii) to assess how age structure of women, child mortality, women's socioeconomic status, and marital patterns interact to influence fertility differentials across districts.<br><br>Methods<br><br>Data were drawn from the 2022 Botswana Population and Housing Census, focusing on women aged 15-49. Fertility estimates were generated using direct methods based on age-specific fertility rates, supplemented by indirect estimation techniques (Rele (1967), Hauer et al. 2013, Ponnapalli & Soren (2018), and Hauer & Schmertmann (2020)) to validate results. Data quality was assessed through analysis of parity and recent births, accounting for potential biases such as recall error, proxy reporting, and reference-period misclassification. Descriptive analysis examined the effects of age structure of women, child mortality, women's socioeconomic status, and marital patterns on fertility variation across districts.<br><br>Results<br><br>The age structure of women revealed a youthful reproductive population, with fertility peaking in the 20-29 age group before declining steadily after age 30. National-level fertility estimates ranged between 2.6 and 3.0 children per woman, consistent across direct and indirect methods, confirming Botswana's advanced stage of fertility transition. However, significant district-level disparities persist. Urban districts (e.g., Gaborone, Francistown, Jwaneng) recorded below-replacement fertility, possibly driven by higher female education, delayed childbearing, and labour market participation. Rural districts (e.g., Ngamiland West, Barolong, Central Mahalapye) maintained higher fertility (3.5-4.5), reflecting elevated child mortality, limited women's empowerment, and traditional reproductive norms. Transitional districts displayed intermediate patterns. Mortality and socioeconomic inequalities were key drivers of

Powered by Editorial Manager® and ProduXion Manager® from Aries Systems Corporation

	spatial fertility variation.<br><br>Conclusion<br><br>This study highlights the reliability of census-based approaches for estimating subnational fertility and underscores the persistent spatial–socioeconomic divides shaping Botswana's demographic landscape. Policy responses must be context-specific: rural districts require strengthened child health, female education, and reproductive health interventions to accelerate fertility transition, while urban centres must prepare for the socioeconomic consequences of sub-replacement fertility. Addressing these divides will be central to ensuring equitable demographic and developmental outcomes in Botswana.
Order of Authors:	Tiro Theodore Monamo, MA
	Kannan Navaneetham, PhD
Opposed Reviewers:	
Additional Information:	
Question	Response
**Financial Disclosure**<br><br>Enter a financial disclosure statement that describes the sources of funding for the work included in this submission. Review the submission guidelines for detailed requirements. View published research articles from *PLOS ONE* for specific examples.<br><br>This statement is required for submission and **will appear in the published article** if the submission is accepted. Please make sure it is accurate.<br><br>**Funded studies**<br>Enter a statement with the following details:<br>• Initials of the authors who received each award<br>• Grant numbers awarded to each author<br>• The full name of each funder<br>• URL of each funder website<br>• Did the sponsors or funders play any role in the study design, data collection and analysis, decision to publish, or preparation of the manuscript?<br><br>Did you receive funding for this work?	The author(s) received no specific funding for this work.
**Competing Interests**<br><br>Use the instructions below to enter a competing interest statement for this	The authors have declared that no competing interests exist

submission. On behalf of all authors, disclose any competing interests that could be perceived to bias this work—acknowledging all financial support and any other relevant financial or non-financial competing interests.<br><br>This statement is required for submission and **will appear in the published article** if the submission is accepted. Please make sure it is accurate and that any funding sources listed in your Funding Information later in the submission form are also declared in your Financial Disclosure statement.<br><br>View published research articles from *PLOS ONE* for specific examples.<br><br>**NO authors have competing interests**<br><br>Enter: *The authors have declared that no competing interests exist.*<br><br>**Authors with competing interests**<br><br>Enter competing interest details beginning with this statement:<br><br>*I have read the journal's policy and the authors of this manuscript have the following competing interests: [insert competing interests here]*<br><br>* typeset	
**Ethics Statement**<br><br>Enter an ethics statement for this submission. This statement is required if the study involved:<br><br>• Human participants<br>• Human specimens or tissue<br>• Vertebrate animals or cephalopods<br>• Vertebrate embryos or tissues<br>• Field research<br><br>Write "N/A" if the submission does not	This paper is based on the analysis of secondary data. Ethical considerations were addressed during the original data collection phases of the census conducted by Statistics Botswana. As per the Botswana Statistics Act of 2009, participation in national surveys and censuses is mandatory, and individuals were informed of this requirement. Additionally, the Act stipulates that refusing to answer any questions constitutes an offence. Consequently, no separate ethical consent process was undertaken for this study, as the necessary ethical protocols were adhered to during the initial data collection.

Powered by Editorial Manager® and ProduXion Manager® from Aries Systems Corporation

require an ethics statement.<br><br>General guidance is provided below. Consult the submission guidelines for detailed instructions. **Make sure that all information entered here is included in the Methods section of the manuscript.**<br><br>**Format for specific study types**<br><br>**Human Subject Research (involving human participants and/or tissue)**<br>• Give the name of the institutional review board or ethics committee that approved the study<br>• Include the approval number and/or a statement indicating approval of this research<br>• Indicate the form of consent obtained (written/oral) or the reason that consent was not obtained (e.g. the data were analyzed anonymously)<br><br>**Animal Research (involving vertebrate animals, embryos or tissues)**<br>• Provide the name of the Institutional Animal Care and Use Committee (IACUC) or other relevant ethics board that reviewed the study protocol, and indicate whether they approved this research or granted a formal waiver of ethical approval<br>• Include an approval number if one was obtained<br>• If the study involved *non-human primates*, add *additional details* about animal welfare and steps taken to ameliorate suffering<br>• If anesthesia, euthanasia, or any kind of animal sacrifice is part of the study, include briefly which substances and/or methods were applied<br><br>**Field Research**<br><br>Include the following details if this study involves the collection of plant, animal, or other materials from a natural setting:<br>• Field permit number<br>• Name of the institution or relevant body that granted permission	
**Data Availability**	Yes - all data are fully available without restriction

Powered by Editorial Manager® and ProduXion Manager® from Aries Systems Corporation

Authors are required to make all data underlying the findings described fully available, without restriction, and from the time of publication. PLOS allows rare exceptions to address legal and ethical concerns. See the PLOS Data Policy and FAQ for detailed information.<br><br>A Data Availability Statement describing where the data can be found is required at submission. Your answers to this question constitute the Data Availability Statement and **will be published in the article**, if accepted.<br><br>**Important:** Stating 'data available on request from the author' is not sufficient. If your data are only available upon request, select 'No' for the first question and explain your exceptional situation in the text box.<br><br>Do the authors confirm that all data underlying the findings described in their manuscript are fully available without restriction?	
**Describe where the data may be found in full sentences. If you are copying our sample text, replace any instances of XXX with the appropriate details.**<br><br>• If the data are **held or will be held in a public repository**, include URLs, accession numbers or DOIs. If this information will only be available after acceptance, indicate this by ticking the box below. For example: *All XXX files are available from the XXX database (accession number(s) XXX, XXX.*).<br>• If the data are all contained **within the manuscript and/or Supporting Information files**, enter the following: *All relevant data are within the manuscript and its Supporting Information files.*<br>• If neither of these applies but you are able to provide **details of access elsewhere**, with or without limitations, please do so. For example:	The data supporting the findings of this study are accessible through Statistics Botswana and can be obtained by researchers upon request. Additionally, the data may be provided by the authors upon reasonable request, subject to approval from Statistics Botswana.

Powered by Editorial Manager® and ProduXion Manager® from Aries Systems Corporation

Data cannot be shared publicly because of [XXX]. Data are available from the XXX Institutional Data Access / Ethics Committee (contact via XXX) for researchers who meet the criteria for access to confidential data.<br><br>*The data underlying the results presented in the study are available from (include the name of the third party and contact information or URL).*<br>• This text is appropriate if the data are owned by a third party and authors do not have permission to share the data.<br><br>* typeset	
Additional data availability information:	

Powered by Editorial Manager® and ProduXion Manager® from Aries Systems Corporation

Tiro Theodore Monamo
P.O. Box 1681, Serowe, Botswana
Email: mtirotheodore@gmail.com
Phone: +267 72535558 / 77885570

15 October 2025

Editor-in-Chief
PLOS ONE

Subject: Submission of Manuscript entitled *"Estimation of District-Level Fertility using Age-Structured Census Data and Assessment of Spatial–Socioeconomic Differentials in Botswana, 2022"*

Dear Editor,

I am pleased to submit our manuscript entitled "Estimation of District-Level Fertility using Age-Structured Census Data and Assessment of Spatial–Socioeconomic Differentials in Botswana, 2022" for consideration for publication in *PLOS ONE*.

This study presents an advanced methodological approach for estimating subnational fertility levels from age-structured census data, validated through established indirect estimation techniques. It provides novel insights into how the age structure of women, child mortality, women's socioeconomic status, and marital patterns interact to produce district-level fertility differentials in Botswana. The results not only confirm the country's continued fertility transition but also reveal the persistence of spatial–socioeconomic divides that have implications for policy and equitable development.

Our work aligns closely with *PLOS ONE*'s mission to disseminate methodologically rigorous, openly accessible, and policy-relevant research across disciplines. Specifically, it contributes to the journal's thematic areas of:

- Demography and Population Studies,
- Quantitative Methods and Statistical Modelling, and
- Public Health and Socioeconomic Inequality Analysis.

This manuscript is original, has not been published elsewhere, and is not under consideration by any other journal. All authors have read and approved the final version of the manuscript, and there are no conflicts of interest to declare.

We believe that this paper will be of interest to readers working in population studies, public health, spatial demography, and policy evaluation, particularly those focusing on fertility dynamics in sub-Saharan Africa.

Thank you for considering our submission. We greatly appreciate your time and look forward to your editorial assessment.

Sincerely,

Tiro Theodore Monamo
P.O. Box 1681, Serowe, Botswana
Email: mtirotheodore@gmail.com
Phone: +267 72535558 / 77885570

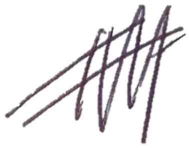

Tiro Theodore Monamo

Manuscript

Click here to access/download;Manuscript;Advanced Techniques for Estimating Fertility.docx

Estimation of District-Level Fertility using Age-Structured Census Data and Assessment of Spatial–Socioeconomic Differentials in Botswana, 2022

Authors and Affiliations

Department of Population Studies, University of Botswana, Private Bag, 00705, Gaborone, Botswana

Tiro Theodore Monamo, Kannan Navaneetham

Corresponding author:

Department of Population Studies, University of Botswana, Private Bag, 00705, Gaborone, Botswana

Tiro Theodore Monamo – mtirotheodore@gmail.com

Abstract

Background: Reliable subnational fertility estimates are essential for guiding demographic policy and planning. In Botswana, national fertility decline has been well documented, but less is known about district-level variation and the socioeconomic factors underpinning it. This study pursued three objectives: (i) to estimate district-level fertility from age-structured census data, (ii) to validate these estimates against established indirect or model-based techniques, and (iii) to assess how age structure of women, child mortality, women's socioeconomic status, and marital patterns interact to influence fertility differentials across districts.

Methods: Data were drawn from the 2022 Botswana Population and Housing Census, focusing on women aged 15-49. Fertility estimates were generated using direct methods based on age-specific fertility rates, supplemented by indirect estimation techniques (Rele (1967), Hauer et al. 2013, Ponnapalli & Soren (2018), and Hauer & Schmertmann (2020)) to validate results. Data quality was assessed through analysis of parity and recent births, accounting for potential biases such as recall error, proxy reporting, and reference-period misclassification. Descriptive analysis examined the effects of age structure of women, child mortality, women's socioeconomic status, and marital patterns on fertility variation across districts.

Results: The age structure of women revealed a youthful reproductive population, with fertility peaking in the 20-29 age group before declining steadily after age 30. National-level fertility estimates ranged between 2.6 and 3.0 children per woman, consistent across direct and indirect methods, confirming Botswana's advanced stage of fertility transition. However, significant district-level disparities persist. Urban districts (e.g., Gaborone, Francistown, Jwaneng) recorded below-replacement fertility, possibly driven by higher female education, delayed childbearing, and labour market participation. Rural districts (e.g., Ngamiland West, Barolong, Central Mahalapye) maintained higher fertility (3.5-4.5), reflecting elevated child mortality, limited women's empowerment, and traditional reproductive norms. Transitional districts displayed intermediate patterns. Mortality and socioeconomic inequalities were key drivers of spatial fertility variation.

Conclusion: This study highlights the reliability of census-based approaches for estimating subnational fertility and underscores the persistent spatial–socioeconomic divides shaping Botswana's demographic landscape. Policy responses must be context-specific: rural districts require strengthened child health, female education, and reproductive health interventions to accelerate fertility transition, while urban centres must prepare for the socioeconomic consequences of sub-replacement fertility. Addressing these divides will be central to ensuring equitable demographic and developmental outcomes in Botswana.

1.0 Introduction

Fertility remains a central driver of demographic change, shaping population structure, influencing the demand for social services, and determining long-term economic trajectories [1]. Reliable measurement of fertility at subnational levels is therefore critical for policy formulation in health, education, labour planning, and social development. Census age-structured data provide a valuable basis for direct estimation of age-specific fertility rates (ASFRs) and the Total Fertility Rate (TFR). However, in many contexts, such direct estimates are rarely validated against alternative model-based or indirect approaches that incorporate demographic adjustments, smoothing techniques, and spatial priors. This limitation has implications for accuracy and comparability, particularly in countries where fertility levels and patterns display marked geographic variation.

Botswana provides an instructive case study in this regard. The country maintains a relatively advanced census system capable of generating district-level data on age and birth distributions [2]. These data capture substantial within-country heterogeneity, ranging from low fertility levels in urban centres to persistently high rates in rural districts [3]. Nevertheless, the direct estimation of fertility from census data is not without challenges. Incomplete birth reporting, age misstatement, in-migration of women of reproductive age, double-counting of births, and cultural or regional reporting biases may distort estimates [4]. In small districts, where recorded births are often minimal, statistical instability further complicates the computation of reliable fertility measures. Moreover, assumptions of uniform mortality and recall period biases can exaggerate fertility estimates, underscoring the need for careful methodological triangulation.

Against this backdrop, the objectives of this paper are threefold. First, it seeks to generate transparent, district-level TFR estimates from the 2022 Botswana census using standard demographic procedures. Second, it compares these direct estimates with model-based fertility measures derived from indirect approaches, including Rele's methods, as well as the frameworks advanced by Hauer and colleagues, Ponnapalli and Soren, and Hauer and Schmertmann. These approaches typically rely on census-based indicators such as child–woman ratios (CWR), interpreting the number of young children per woman in defined age groups as a proxy for fertility levels in the absence of complete vital registration data. Third, the study investigates spatial and socioeconomic fertility differentials across districts, examining links with covariates such as female employment, under-five mortality, age structure, marital and cohabitation status, and tertiary education attainment.

The contribution of this study is both methodological and substantive. Methodologically, it demonstrates the value of rigorous direct estimation, while embedding results within a transparent comparative framework that integrates established model-based approaches. Substantively, it provides empirical evidence on the drivers of fertility variation across Botswana's districts, highlighting the influence of socioeconomic and spatial determinants. The policy relevance is immediate: accurate and reliable district-level fertility estimates are indispensable for guiding reproductive health services, educational programming, and family planning interventions tailored to local needs [5].

The demographic context in Botswana underscores the importance of such an endeavour. Fertility in the country has undergone a pronounced transition over the past five decades. Research by Bainame and Letamo [6], which employed the Arriaga method to estimate fertility trends between 1971 and 2011, documented a sustained decline from 6.6 children per woman in 1981 to 3.3 in 2001, and further to 2.8 by 2011. These shifts reflected broader socioeconomic transformations, including urbanization, rising educational attainment, and increased participation of women in the labour force, factors consistently associated with lower fertility, particularly in urban areas [7, 8]. Nonetheless, national-level analyses obscure regional and district-level differences that are essential for a nuanced understanding of fertility dynamics.

Despite the availability of high-quality census data, fertility studies at disaggregated spatial scales remain limited in Botswana. This gap restricts the ability of policymakers to design context-specific interventions, particularly in rural and remote districts where fertility rates and demographic behaviours diverge from national averages. Furthermore, the limited application of indirect and model-based estimation techniques at the district level hampers the validation of direct measures, raising questions about the reliability of estimates used in planning. Addressing these gaps, the present study not only produces direct census-based TFR estimates but also systematically evaluates them against model-derived measures and explores their socioeconomic determinants, offering a comprehensive framework for understanding fertility patterns in Botswana at a level of granularity critical for policy and planning.

2.0 Data and Methods

2.1 Data Source

This study draws on data from the 2022 Botswana Population and Housing Census (PHC), the sixth national census conducted by Statistics Botswana. The census, undertaken every ten years, provides the most comprehensive source of demographic and socioeconomic information for the country, and serves as the primary data source for district-level fertility estimation in this paper. A total of 2,346,179 individuals were enumerated [9], with enumeration based on de facto residence, meaning individuals were recorded where they were found during the census period. The census also captured detailed information on births, household characteristics, educational attainment, employment, and marital status, making it well-suited for fertility analysis and for examining socioeconomic determinants of demographic outcomes.

The 2022 PHC introduced major methodological innovations by being the first census in Botswana to fully adopt digital technologies, from cartographic mapping to household enumeration [10]. This transition enhanced both the speed and quality of data collection, as evidenced by improvements identified during preliminary data edits and the accelerated release of census results. The census reported a national average household size of 3.3 persons, a decline from 3.7 recorded in 2011, reflecting ongoing demographic transition. For the purposes of this study, fertility measures were derived from age-structured population counts of women aged 15-49 and children under the age of five, while complementary variables such as employment, education, and marital status were extracted to examine spatial–socioeconomic fertility differentials across districts. These features make the 2022 census an indispensable and reliable foundation for analysing fertility levels, trends, and differentials in Botswana.

2.2 Evaluation of Fertility Data Quality

2.2.1 Assessment of parity data

In population censuses, the first question on fertility typically concerns women's lifetime fertility, measured by the number of children ever born [4]. As with most self-reported demographic data, fertility information is subject to several potential reporting errors. One important issue is recall bias, which tends to increase with the respondent's age. Older women are more likely to omit births, particularly those that occurred early in their reproductive lives or children who subsequently died [11]. A second concern arises when fertility data are provided by proxy respondents rather than the mother herself. In such cases, the proxy may have incomplete knowledge of the woman's reproductive history, especially regarding children who reside elsewhere or those who have died. Both of these factors may lead to the under-enumeration of parities.

Despite these potential challenges, the 2022 Botswana Population and Housing Census parity data display an overall pattern that aligns with demographic expectations. **Figure 1** illustrates a positive association between women's age and average parity**,** whereby the number of children ever born increases steadily across age groups. This age gradient is consistent with biological and demographic realities, since older women, having had longer exposure to

childbearing, report higher parities than younger cohorts. The observed trend supports the general reliability of the parity data as a basis for estimating district-level fertility in Botswana.

Figure 1: Distribution of women by age and parity

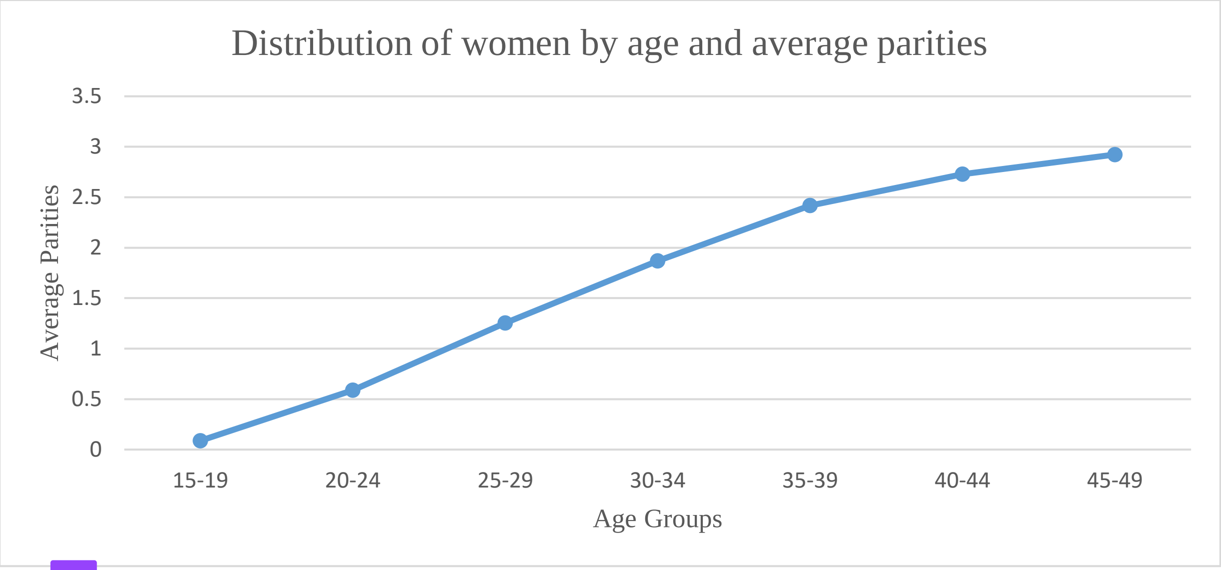

2.2.2 Assessment of Recent Births Data

In addition to parity, the quality of data on recent births is critical for estimating fertility levels and patterns. Several potential sources of error may affect the accuracy of this information. First, there is uncertainty regarding the exact timing of births in relation to the census reference period. This uncertainty may lead to the misplacement of births, either by incorrectly including births that occurred before the reference period or excluding those that occurred within it. Such reference-period misclassifications can bias fertility estimates.

Second, migration and mortality dynamics may contribute to under-reporting. For instance, women who had a recent birth but subsequently died or migrated out of the household prior to enumeration will not be captured in the census data. Similarly, households that experienced dissolution after a birth occurred may also fail to report these events. These omissions result in an underestimation of recent fertility levels.

Despite these challenges, the reported age-specific fertility pattern in Botswana's 2022 Census data follows the expected demographic trajectory. As shown in **Figure 3**, fertility rises steadily from ages 15 to 29**,** reflecting the concentration of childbearing in early adulthood. Fertility then declines progressively from ages 30 to 49, consistent with biological limits on fecundity and sociocultural shifts that typically accompany later reproductive ages. This pattern mirrors global evidence: for example, estimates from the American Society for Reproductive Medicine indicate that women under age 30 have approximately a 25% probability of conceiving naturally per cycle, declining to about 20% after age 30, and further reducing to around 5% by age 40 [12].

The alignment of the Botswana census results with established demographic expectations provides reassurance regarding the overall reliability of recent births data, despite the known limitations associated with recall, reporting, and household dynamics

Figure 2: Distribution of women by age and observed ASFRs

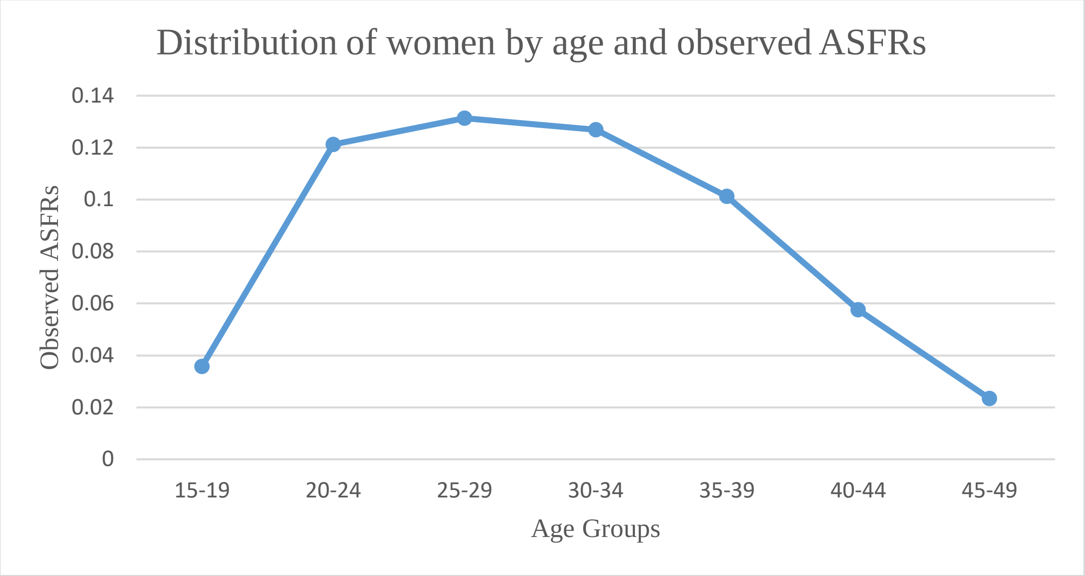

2.3 Methodological Framework for Estimating District-Level Fertility

This study employed both direct and indirect approaches to estimate fertility levels at the district level in Botswana. The direct estimation was undertaken by computing Age-Specific Fertility Rates (ASFRs) for women aged 15-49, grouped into conventional five-year intervals. The Total Fertility Rate (TFR) was then derived by summing the ASFRs across all age groups, representing the average number of children a woman would bear if exposed to current age-specific fertility patterns throughout her reproductive life. While this method is transparent and widely used in demographic research, it is also sensitive to census data quality, particularly completeness of birth reporting, accuracy of women's age distribution, and district-level population mobility. For this reason, the study triangulates these direct estimates with several established indirect techniques.

Among the indirect approaches, Rele's method [13] was applied as one of the foundational techniques in fertility estimation. This approach relies on the Child–Woman Ratio (CWR), calculated as the number of children aged 0–4 per 1,000 women of reproductive age (15–49). The method exploits the statistical relationship between the Gross Reproduction Rate (GRR) and the CWR in stable populations, adjusted for mortality using coefficients (α and β) corresponding to different levels of life expectancy. The TFR is then approximated as 2.05 times the GRR. Rele's method is particularly valuable in data-limited settings due to its minimal requirements and capacity to produce fertility estimates over two periods, using children aged 0–4 to represent births in the 2.5 years preceding the census, and children aged 5–9 for births in the preceding 7.5 years. Despite assumptions of population stability and

uniform mortality, this approach has been widely validated in diverse contexts and provides a useful comparative benchmark for the Botswana data.

A second set of indirect estimates was derived using the Child–Woman Ratio approach advanced by Hauer et al. [14]. This method builds on the established empirical relationship between the General Fertility Rate (GFR) and the TFR, simplifying estimation by treating the reproductive span as a single interval. The implied TFR (iTFR) is calculated using the number of children aged 0–4 relative to women of childbearing age, thereby reducing data demands. The method's strength lies in its feasibility in low-data environments and its adaptability to census structures where detailed fertility histories are unavailable. Similarly, the technique proposed by Ponnapalli and Soren [15] was implemented, extending the use of CWRs to estimate not only TFR but also related measures such as Crude Birth Rate (CBR), General Fertility Rate (GFR), and marital fertility indicators. This approach incorporates child populations aged 0–9, total female populations, and marital status data, making it especially suitable for subnational analyses where vital registration is incomplete. Its validation in the Indian census context underscores its robustness, and its minimal data requirements align well with Botswana's district-level demographic records.

Finally, the study applied the more recent methodological framework developed by Hauer and Schmertmann [16], which refines indirect fertility estimation by incorporating demographic information embedded in population pyramids. Their Extended Total Fertility Rate (xTFR) model adjusts traditional child–woman ratio approaches by accounting for the proportion of women concentrated in high-fertility age groups (25–34) relative to the broader reproductive span, thereby improving accuracy in populations with non-uniform age structures. Moreover, the adjusted xTFR+ variant integrates under-five mortality (q5) through a survival multiplier, correcting biases that arise in settings where child mortality remains significant. Given the observed fluctuations in Botswana's under-five mortality over the past decade, the xTFR+ provides an important correction to conventional indirect estimates. By employing this suite of direct and indirect approaches, the study establishes a comprehensive and comparative framework for estimating district-level fertility, allowing for robust validation of census-based measures and deeper insights into spatial–socioeconomic fertility differentials.

The integration of both direct and model-based estimation techniques is essential for ensuring that fertility measures used in planning are not only methodologically sound but also contextually reliable. District-level variations in Botswana reflect complex interactions between data quality, demographic behaviours, and socioeconomic conditions. By triangulating direct estimates with multiple indirect approaches, the study minimizes the risk of systematic bias and provides policymakers with more dependable fertility indicators. This methodological rigour enhances the utility of the findings for designing targeted reproductive health services, education programmes, and labour market policies that are sensitive to the distinct demographic realities of each district.

2.4 Measurement of the Validity of District-Level Fertility Estimates

To assess the validity of the direct district-level TFR estimates, a comparative validation exercise was undertaken against five established model-based fertility measures. For each district, pairwise comparisons were made between the direct census estimates and the corresponding model-based estimates. The strength of association was quantified using Pearson correlation coefficients, enabling an assessment of the degree of linear correspondence between the direct and indirect measures. Correlation values were interpreted in line with conventional thresholds, with coefficients ranging from 0.70 to 0.89 considered strong and those above 0.90 interpreted as very strong alignment [17]. In addition to correlation analysis, descriptive comparisons were performed to identify systematic differences across districts, with particular attention to deviations in urban versus rural settings. This combined approach allowed for both statistical validation of the direct estimates and substantive interpretation of areas where divergences may reflect underlying demographic or socioeconomic processes rather than methodological error.

2.5 Assessing the Effects of Mortality, Women's Socioeconomic Status, and Marital Patterns on Fertility

To examine the determinants of district-level fertility variation in Botswana, the study linked direct Total Fertility Rate (TFR) estimates from the 2022 census with district-level indicators of child mortality, women's socioeconomic status, and marital patterns. Mortality was measured using infant and under-five mortality rates (U5MR) obtained from census data, providing a proxy for survival risks that may influence fertility through replacement behaviour. Women's socioeconomic status was captured through two key variables: the proportion of women aged 15–49 with tertiary education and the proportion of women employed, both derived from census microdata. Marital patterns were operationalized as the percentage of women aged 15–49 who were married or cohabiting in each district. Particular attention was given to urban–rural contrasts, transitional districts, and outliers, in order to understand how interactions between mortality, education, employment, and marital behaviour shape fertility outcomes. This integrated approach allowed the study to situate district-level fertility patterns within a broader demographic transition framework while highlighting context-specific dynamics unique to Botswana.

Ethics Considerations

This paper is based on the analysis of secondary data. Ethical considerations were addressed during the original data collection phases of the census conducted by Statistics Botswana. As per the Botswana Statistics Act of 2009, participation in national surveys and censuses is mandatory, and individuals were informed of this requirement. Additionally, the Act stipulates that refusing to answer any questions constitutes an offence. Consequently, no separate ethical consent process was undertaken for this study, as the necessary ethical protocols were adhered to during the initial data collection.

3.0 Findings

This section presents the results of the study in four interrelated parts. First, it provides a comparative analysis of district-level fertility estimates derived from both direct census-based measures and a range of indirect demographic models, highlighting areas of convergence and divergence. Second, it evaluates the validity of these estimates by examining their consistency across methods and assessing the reliability of census-derived fertility indicators. Third, the section explores the influence of women's age structure on fertility outcomes, assessing how differences in the distribution of reproductive-age cohorts shape district-level patterns. Finally, it investigates the intersecting effects of child mortality, women's socioeconomic status, and marital dynamics in driving regional fertility divergences.

3.1 Comparative Analysis of District-Level Fertility Estimates

The district-level fertility estimates presented in **Table 1** highlight both consistencies and divergences across direct and indirect estimation techniques, underscoring the complexity of measuring fertility at subnational scales in Botswana. Across all districts, the direct estimates derived from the 2022 census generally align closely with model-based outputs, with national-level totals ranging from 2.6 to 3.0 across indirect methods and 2.9 from the direct approach. This convergence provides strong validation for the robustness of the 2022 census estimates, while also revealing systematic patterns of variation that reflect underlying demographic and socioeconomic contexts.

Urban districts consistently record the lowest fertility levels, regardless of the method applied. For instance, Gaborone City's direct estimate of 1.8 children per woman closely matches indirect estimates (1.5–1.7), placing the capital well below replacement level fertility. Similar patterns are evident in Francistown (2.2), Jwaneng (1.8), and Sowa Town (1.4), where direct measures corroborate the persistent fertility suppression in urban and mining towns. These low values are reflective of broader demographic transitions, including delayed childbearing, higher levels of female education, and stronger participation of women in the labour market. Notably, however, small differences emerge across methods; for example, Sowa Town's direct estimate (1.4) is substantially lower than model-based outputs (2.0–2.9), likely reflecting data volatility in very small populations.

By contrast, rural districts show persistently high fertility, often exceeding 3.5 children per woman. Districts such as Ngamiland West (direct estimate 4.5), Central Tutume (3.7), Barolong (3.9), and Kweneng West (3.7) exemplify this pattern. Here, indirect estimates are generally consistent but occasionally understate fertility, suggesting that model-based approaches may not fully capture the reproductive intensity of rural populations. For example, Ngamiland West's direct estimate (4.5) is higher than any indirect method (3.8–4.4), highlighting the potential for census-based direct estimates to detect fertility peaks not fully anticipated by models. These divergences underline the importance of triangulating multiple approaches, particularly in rural contexts where incomplete reporting, higher child mortality, and limited family planning uptake interact to sustain high fertility.

Intermediate fertility levels are observed in semi-urban and peri-urban districts such as Kgatleng (2.9), Kweneng East (2.9), and Serowe/Palapye (3.1). These values fall between the extremes of urban and rural fertility, reflecting transitional demographic regimes where urbanization and education exert downward pressures, while traditional reproductive behaviours remain influential. Across these districts, the agreement between direct and indirect estimates is generally strong, with variations rarely exceeding 0.4 children per woman. At the national level, the convergence between direct (2.9) and model-based estimates (2.6–3.0) confirms that Botswana remains in the midst of its fertility transition, with district-level heterogeneity reflecting uneven progress across regions.

Overall, the table illustrates that while indirect methods remain valuable for comparative and historical analyses, direct estimates derived from recent census data capture emerging fertility dynamics with greater sensitivity, especially in rapidly urbanizing or persistently high-fertility rural districts. These findings reinforce the utility of combining methods to achieve both accuracy and comparability in fertility estimation.

Table 1: Comparative District-Level Fertility Estimates in Botswana, 2014–2022: Direct Census-Based and Indirect Model-Based Methods

District	Rele TFR-1 2019 Estimates	Rele TFR-2 2014 Estimates	Hauer et al. (2013) 2022 Estimates	Ponnapalli & Soren (2018) 2022 Estimates	Hauer & Schmertmann (2020) 2022 Estimates	Direct Estimates (2022)
**Gaborone City**	1.5	1.7	1.6	1.5	1.5	1.8
**Francistown City**	2.3	2.4	2.3	2.1	2.1	2.2
**Lobatse**	2.2	2.6	2.1	2.1	2.1	2.5
**Selebi Pikwe**	2.3	3.1	2.2	2.4	2.3	2.3
**Orapa**	1.9	3.0	1.9	2.2	2.0	2.6
**Jwaneng**	1.8	2.4	1.8	1.9	1.7	1.8
**Sowa Town**	2.1	2.9	2.1	2.3	2.0	1.4
**Barolong**	3.8	4.1	3.4	3.4	3.6	3.9
**Ngwaketse West**	3.9	4.0	3.5	3.5	3.7	3.6
**South East**	1.8	1.9	1.9	1.7	1.7	2.2
**Kweneng East**	2.5	2.5	2.5	2.2	2.3	2.9
**Kweneng West**	4.0	4.0	3.7	3.6	3.9	3.7
**Kgatleng**	2.7	2.7	2.6	2.4	2.6	2.9
**Serowe Palapye**	3.3	3.5	3.1	3.0	3.2	3.1
**Central Mahalapye**	3.9	4.0	3.5	3.4	3.8	3.4
**Central Bobonong**	3.9	4.1	3.6	3.5	3.8	3.4
**Central Boteti**	3.4	3.7	3.3	3.2	3.2	3.6
**Central Tutume**	3.8	4.1	3.5	3.5	3.7	3.7
**North East**	3.5	3.8	3.2	3.2	3.4	3.1
**Ngamiland East**	3.2	3.3	3.1	2.9	2.9	3.3
**Ngamiland West**	4.4	4.0	4.1	3.8	4.1	4.5
**Chobe**	2.5	2.4	2.5	2.3	2.2	3.2
**Ghanzi**	3.1	3.1	3.0	2.8	2.8	3.2
**Kgalagadi South**	3.3	3.6	3.2	3.0	3.2	3.0
**Kgalagadi North**	2.9	3.0	2.9	2.7	2.7	2.8
**Total**	**2.8**	**3.0**	**2.7**	**2.6**	**2.7**	**2.9**

3.2 Validation of District-Level Fertility Estimates

Validation of the district-level fertility estimates was undertaken by comparing the direct estimates derived from the 2022 Botswana Population and Housing Census with a set of established model-based estimates, namely the Rele TFR-1, Rele TFR-2, Hauer et al. (2013), Ponnapalli and Soren (2018), and Hauer and Schmertmann (2020)[illegible]he results reveal a generally strong alignment across measures, with correlation coefficients ranging from 0.78 to 0.91. The strongest agreement was observed between the direct census estimates and Rele TFR-1 (2019) ($r \approx 0.90$) and Hauer et al. (2013) ($r \approx 0.91$), indicating that both recent indirect census adjustments and longer-term model-based projections captured the prevailing fertility patterns with considerable accuracy. Strong correlations were also observed with Ponnapalli and Soren (2018) ($r \approx 0.87$) and Hauer and Schmertmann (2020) ($r \approx 0.88$), further validating the internal consistency of the 2022 direct estimates. The weakest association but still strong, emerged with Rele TFR-2 (2014) ($r \approx 0.78$), reflecting the temporal gap and the continuing fertility transition over the past decade, particularly the accelerated fertility decline in urban and peri-urban districts such as Gaborone, South-East, and Lobatse.

Importantly, the divergences observed across methods do not undermine the credibility of the 2022 census direct estimates; rather, they reflect real demographic shifts occurring over time. Urban districts consistently exhibit lower fertility than anticipated by earlier models, a trend consistent with rising female educational attainment, increased labour market participation, and delayed or less frequent marriage and cohabitation. Conversely, higher-fertility rural districts such as Ngamiland West, Barolong, and Central Mahalapye display direct estimates that slightly exceed model-based predictions, an outcome plausibly linked to persistent socioeconomic disadvantage, higher child mortality, and weaker diffusion of fertility-limiting behaviours. These findings underscore the robustness of the direct TFRs, which both validate and extend model-based estimates by capturing the most recent dynamics of Botswana's fertility transition. Taken together, the strength of these correlations supports the use of the 2022 direct estimates as the primary basis for subsequent analysis of spatial and socioeconomic fertility differentials, with the model-based measures serving as complementary benchmarks that enhance confidence in the observed patterns.

Figur[illegible] Correlation Between Direct 2022 Census-Based Fertility Estimates and Model-Based Estimates at the District Level in Botswana

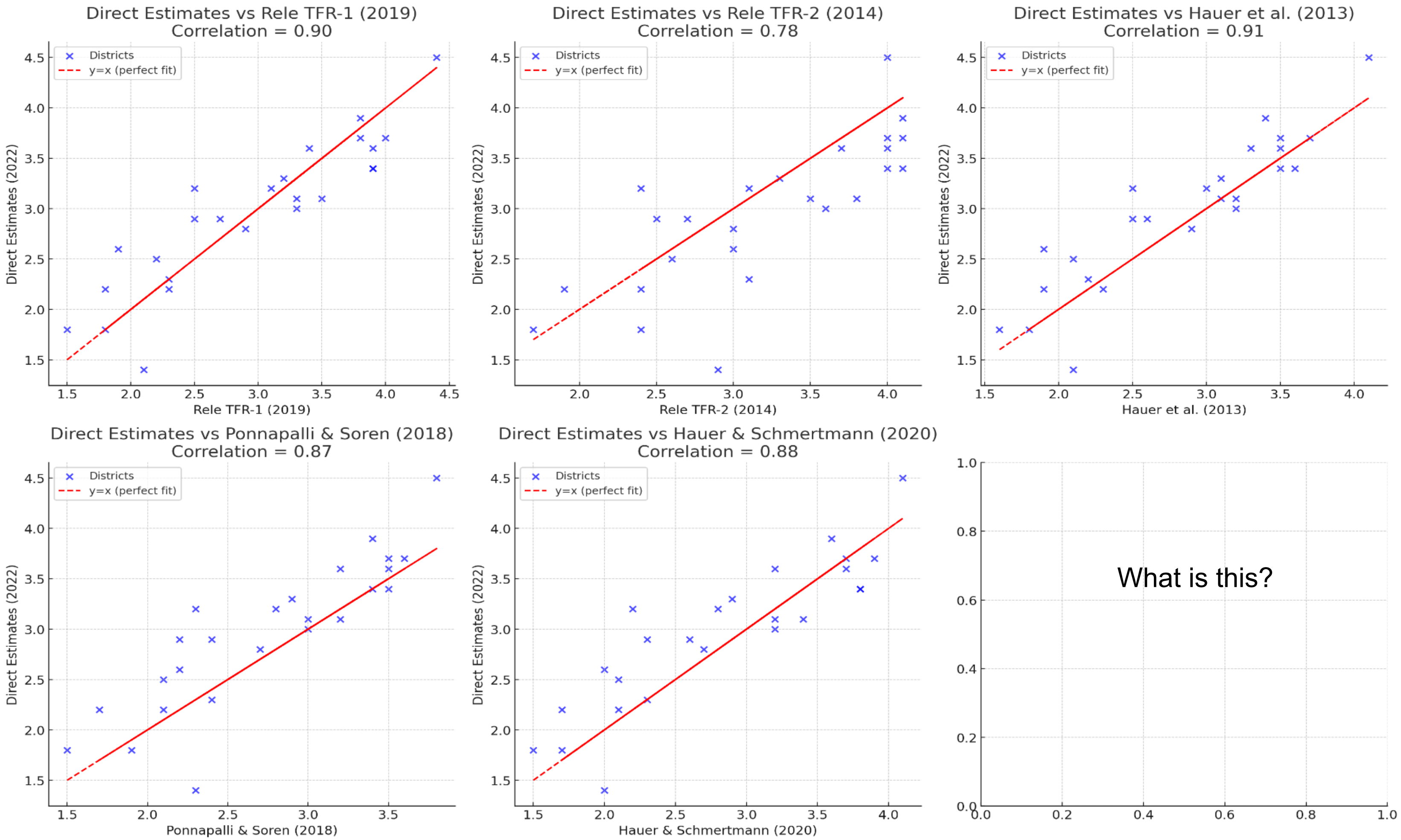

3.3 Age Structure of Women of Reproductive Age and Its Influence on District-Level Fertility in Botswana

The distribution of women across reproductive age groups strongly shapes the observed district-level total fertility rates (TFR) in Botswana. Districts where younger cohorts (15–24) constitute a relatively high proportion of women tend to exhibit elevated fertility levels, as childbearing is concentrated in these age groups. For example, Ngamiland West, which records the highest TFR (4.5), shows substantial representation in the younger reproductive ages (15–29), reflecting early onset of childbearing and limited postponement of fertility. Similarly, Barolong (TFR 3.9) and Central Tutume (3.7) exhibit youthful age structures that amplify fertility outcomes, as a large proportion of women are in the prime childbearing ages of 20–34.

Conversely, urban districts with lower fertility, such as Gaborone (1.8), Jwaneng (1.8), and Sowa Town (1.4), display broader distribution of women across later reproductive ages, with substantial proportions in the 30–44 range. This pattern possibly indicates delayed childbearing, fertility limitation, and increased birth spacing, which suppress total fertility despite large absolute populations of women. In Gaborone, for instance, more than 12,000 women are concentrated in the 30–34 age group and over 10,000 in 40–44, reflecting a demographic profile where reproductive timing is later and more controlled, contributing to below-replacement fertility.

Intermediate districts such as Serowe/Palapye (TFR 3.1) and Kweneng East (2.9) illustrate how the balance between younger and older reproductive cohorts moderates fertility outcomes. While these districts have large populations of women in the 20–34 age range, they also show significant representation in older cohorts (35–44), which dampens overall fertility levels compared to more youthful rural districts.

Taken together, the patterns suggest that district fertility variation in Botswana is closely linked to the relative weight of younger versus older female cohorts. Districts with youthful female populations experience elevated fertility possibly due to early initiation of childbearing and limited fertility control, while those with a more balanced or older reproductive-age distribution register lower fertility as births are delayed, spaced, or curtailed. This reinforces the demographic principle that age structure interacts with social and economic conditions to shape fertility trajectories, positioning urban Botswana firmly within a low-fertility regime while rural districts remain characterized by high fertility driven by youthful reproductive profiles.

Table 2: Age Distribution of Women of Reproductive Age (15–49) and Its Influence on District-Level Total Fertility Rates (TFR) in Botswana

District	15-19	20-24	25-29	30-34	35-39	40-44	45-49	Total (15-49)	TFR
Gaborone	11182	15015	13693	12875	12510	10563	7912	83550	1.8
Francistown	4744	5046	5134	5249	5354	3858	2578	31963	2.2
Lobatse	1302	1165	1353	1336	1427	1221	863	8667	2.5
Selibe Phikwe	2229	1480	1813	1870	2116	1800	1341	12849	2.3
Orapa	352	294	322	449	612	505	327	2861	2.6
Jwaneng	692	697	904	948	1041	810	530	4622	1.8
Sowa Town	111	102	158	128	127	131	84	841	1.4
Ngwaketse West	928	758	865	806	856	644	551	5408	3.6
Barolong	2214	1778	1913	1863	1990	1701	1527	12986	3.9
South-East	4692	6900	6110	5490	5003	4204	3341	35740	2.2
Kweneng East	13917	15772	16249	15104	14540	11913	8699	96194	2.9
Kweneng West	2196	1945	1999	1940	2031	1545	1268	12924	3.7
Kgatleng	5009	4978	5204	4982	4963	4318	3354	32808	2.9
Serowe/Palapye	8480	7242	7698	7494	7641	6512	5086	50153	3.1
Central Mahalapye	5143	3942	4189	4380	4638	4100	3322	29714	3.4
Central Bobonong	3042	2361	2659	2525	2758	2433	2083	17861	3.4
Central Boteti	3004	2745	3208	3143	3111	2322	1571	19104	3.6
Central Tutume	6728	5422	5777	5747	5795	4783	4190	38442	3.7
North-East	2727	2248	2473	2553	2596	2169	1812	16578	3.1
Ngamiland East	5140	4888	5532	5690	5119	3776	2685	31830	3.3
Ngamiland West	3052	2781	3218	2861	2813	2121	1550	18396	4.5
Chobe	881	1177	1554	1477	1417	1037	738	8281	3.2
Ghanzi	2198	2374	2478	2622	2151	1545	1301	14669	3.2
Kgalagadi South	1532	1206	1400	1306	1328	1126	1018	8916	3.0
Kgalagadi North	883	823	1118	930	953	771	565	5043	2.8

3.4 Intersecting Effects of Mortality, Women's Socioeconomic Status, and Marital Patterns in Shaping Regional Fertility Divergence in Botswana

The analysis of district-level fertility patterns in Botswana reveals strong associations between women's socioeconomic status, mortality outcomes, and total fertility rate (TFR). Urban districts such as Gaborone, Francistown, Lobatse, Orapa, Jwaneng, and Sowa Town consistently exhibit low fertility levels (TFR 1.4-2.6), which align with broader indicators of social modernization. These areas are characterized by low infant and under-five mortality, high levels of female employment, and significant attainment of tertiary education. In Gaborone, for instance, nearly half of women have tertiary education (46.3%), and employment levels exceed 50%, contributing to the lowest fertility rates in the country (TFR 1.8). Sowa Town presents an even more striking case, with fertility suppressed to 1.4 despite high rates of marriage or cohabitation (39.5%). This suggests that where women's labour force participation and educational attainment are strong, fertility remains low regardless of union prevalence, consistent with the tenets of the demographic transition theory.

By contrast, rural districts such as Barolong, Ngwaketse West, Central Mahalapye, Central Tutume and Ngamiland West are characterized by high mortality and limited female educational attainment, resulting in elevated fertility levels (TFR 3.4-4.5). For example, Ngamiland West records both the highest child mortality (U5MR 36) and the highest fertility (TFR 4.5), alongside one of the lowest levels of female tertiary education (12.7%). Here, mortality exerts a replacement effect, with families maintaining higher fertility as a strategy against the risk of child loss, while low education and restricted economic opportunities for women reinforce traditional reproductive norms. Similarly, Barolong exhibits high mortality and one of the lowest levels of tertiary education among women (15.4%), correlating with very high fertility (TFR 3.9). These cases illustrate how mortality and low female empowerment perpetuate high fertility in rural Botswana, delaying convergence with urban fertility patterns.

Intermediate districts such as Kweneng East, Kgatleng, Serowe-Palapye, North-East, and Chobe exhibit moderate fertility levels (TFR 2.8-3.3), reflecting transitional dynamics. In these areas, female employment and education are improving but remain uneven, and mortality rates are higher than in urban centres. Chobe provides a noteworthy example: despite high mortality (U5MR 28), fertility is moderated to 3.2, largely due to female employment rates exceeding 50%. This case demonstrates that employment opportunities can act as a fertility-reducing factor, counterbalancing the upward pressure exerted by high mortality. Similarly, in Kweneng East and Kgatleng, moderate fertility (TFR 2.9) reflects an equilibrium between improving education levels and persisting traditional family formation practices.

The patterns observed confirm that fertility in Botswana is shaped by the interaction of mortality dynamics and women's socioeconomic status**.** Districts with low mortality, high education, and high employment invariably demonstrate low fertility, even when marriage rates are moderate to high. Conversely, districts with high mortality and limited educational and employment opportunities for women continue to experience high fertility, consistent with both replacement and demand-side theories of fertility. Transitional districts illustrate that

incremental gains in women's empowerment and labour market participation can mitigate the fertility-raising effects of elevated mortality, highlighting pathways for policy intervention.

Table 1: District-Level Associations Between Mortality, Women's Socioeconomic Characteristics, and Total Fertility Rates (TFR) in Botswana

District	IMR	U5MR	% Women Employed	% Women Married/Cohabiting	% Women Tertiary Education	TFR	Interpretation
Gaborone City	8	9	50.7	30.1	46.3	1.8	Low mortality, high employment and education, low union prevalence → lowest TFR
Francistown City	12	14	48.3	31.4	28.7	2.2	Moderate mortality, high employment, moderate education → low TFR
Lobatse	10	11	48.9	32.0	29.0	2.5	Low mortality, high employment → low fertility
Selebi Phikwe	21	24	46.8	37.2	27.1	2.3	Higher mortality slightly increases TFR, despite moderate employment and education
Orapa	0	4	58.9	40.7	43.4	2.6	Very low mortality, high employment and education → moderate TFR due to migration and family formation dynamics
Jwaneng	18	18	56.2	37.9	39.0	1.8	moderate mortality, high employment/education → low TFR
Sowa Town	0	0	57.8	39.5	39.9	1.4	Extremely low mortality, high employment/education → lowest TFR nationally
Barolong	21	25	32.4	30.5	15.4	3.9	High mortality, low employment and education → very high TFR
Ngwaketse West	24	28	33.3	21.8	12.6	3.6	High mortality, low education/employment → high TFR
South East	8	11	46.6	25.6	41.9	2.2	Low mortality, high employment and education → low TFR
Kweneng East	11	14	36.9	24.1	26.3	2.9	Moderate mortality, moderate education/employment → mid-range TFR

Kweneng West	23	26	32.9	31.7	11.3	3.7	High mortality, low education → high TFR
Kgatleng	12	14	39.4	26.0	26.8	2.9	Moderate mortality and education → moderate TFR
Serowe Palapye	15	19	34.8	20.7	20.8	3.1	Moderate mortality, low employment/education → slightly high TFR
Central Mahalapye	23	28	33.2	20.8	15.6	3.4	High mortality, low education → high TFR
Central Bobonong	28	34	41.7	28.2	16.8	3.4	Very high mortality, moderate employment → high TFR
Central Boteti	14	19	36.2	26.5	18.3	3.6	Moderate mortality, low education → high TFR
Central Tutume	21	25	35.9	24.4	16.1	3.7	High mortality, low education/employment → high TFR
North East	17	18	44.0	34.6	22.3	3.1	Moderate mortality, moderate education/employment → slightly above average TFR
Ngamiland East	18	23	37.9	28.0	23.3	3.3	Moderate mortality, low-medium education → higher TFR
Ngamiland West	29	36	34.8	31.6	12.7	4.5	Highest mortality, low employment/education → highest TFR nationally
Chobe	23	28	54.4	35.9	23.2	3.2	High employment moderates fertility despite high mortality → TFR slightly above national average
Ghanzi	25	33	40.8	36.2	20.1	3.2	High mortality, moderate education/employment → high TFR
Kgalagadi South	22	25	38.3	23.1	15.6	3.0	High mortality, low education → above-average TFR

Kgalagadi North	25	28	48.2	28.2	21.4	2.8	High employment reduces fertility moderately → TFR slightly below rural averages

4.0 Discussion and Conclusion

This study set out to estimate district-level fertility from age-structured census data and assess spatial–socioeconomic differentials in Botswana using the 2022 Population and Housing Census. Three core objectives guided the analysis: (i) to generate transparent district-level Total Fertility Rate (TFR) estimates using direct census methods, (ii) to validate these estimates through comparisons with multiple indirect model-based approaches, and (iii) to examine how mortality, women's socioeconomic status, and marital patterns interact to shape fertility variation across districts.

4.1 Critical Discussion of Findings

The findings demonstrate a robust alignment between direct and indirect estimates, with national-level fertility converging around 2.6–3.0 children per woman, consistent with Botswana's ongoing fertility transition. Importantly, district-level heterogeneity remains pronounced. Urban centres such as Gaborone, Francistown, and mining towns like Jwaneng and Sowa Town record fertility well below replacement, reflecting patterns of delayed childbearing, high female education, and strong labour market participation. Conversely, rural districts such as Ngamiland West, Barolong, and Central Mahalapye exhibit persistently high fertility (3.5–4.5), shaped by elevated child mortality, limited women's empowerment, and adherence to traditional reproductive norms. Transitional districts occupy the middle ground, where education and employment gains moderate but do not eliminate the influence of high mortality and early childbearing.

These results align with previous studies on Botswana's demographic transition. Bainame and Letamo [6] documented a long-term national fertility decline from 6.6 in 1981 to 2.8 in 2011, yet this study provides novel insights by highlighting persistent spatial divides. The patterns are consistent with broader evidence from sub-Saharan Africa and other developing contexts, where fertility transitions are uneven across rural and urban areas [18, 19]**.**In particular, the association of low fertility with women's tertiary education and employment echoes findings from South Africa [20], while the persistence of high fertility in regions of elevated child mortality parallels results from East African settings where replacement behaviour sustains reproductive intensity [21].

A critical contribution of this study lies in its methodological triangulation. By validating direct census estimates against established model-based techniques (Rele, Hauer et al., Ponnapalli & Soren, Hauer & Schmertmann), the analysis provides reassurance regarding the reliability of Botswana's census as a source for subnational fertility monitoring. The strong correlations observed, particularly with Hauer et al. (2013) and Rele (2019), underscore the value of embedding census-based estimation within comparative frameworks. At the same time, divergences in small districts such as Sowa Town highlight the limitations of indirect models in contexts of small populations, where volatility can exaggerate differences.

4.2 Policy Implications

The findings carry significant policy relevance. First, the persistent urban–rural fertility divide calls for targeted interventions that acknowledge regional heterogeneity. In rural districts where fertility remains high, policies should prioritize investments in female education, economic empowerment, and reproductive health services, including improved access to family planning. These measures are likely to accelerate the transition towards smaller family sizes.

Second, the association between child mortality and high fertility highlights the urgency of further reducing under-five mortality through investments in child health, nutrition, and healthcare access. Mortality reduction can break the cycle of replacement behaviour that sustains high fertility in disadvantaged districts.

Third, in urban centres where fertility is below replacement, policymakers must anticipate potential long-term implications, including population ageing, labour force contraction, and rising dependency ratios. This calls for forward-looking strategies such as supporting work–family balance, encouraging sustainable family sizes, and adapting social and economic policies to mitigate demographic shifts.

Finally, the demonstrated utility of census-based fertility estimation underscores the need to institutionalize regular district-level demographic monitoring, integrating direct census estimates with innovative model-based approaches. This would strengthen evidence-based planning in education, health, housing, and labour market development, ensuring interventions are aligned with the demographic realities of each district.

4.3 Limitations

Despite its contributions, the study is not without limitations. First, census-based fertility data remain vulnerable to recall bias, proxy reporting, and reference-period errors, which may understate or misclassify births. Second, small population districts introduce volatility into direct estimates, as observed in Sowa Town. Third, the cross-sectional nature of census data limits causal inference; while associations between fertility and socioeconomic factors are observed, longitudinal data would be needed to confirm directionality and dynamics over time. Finally, indirect methods rely on assumptions of stable populations and uniform mortality, which may not hold in contexts of rapid demographic transition or internal migration.

4.4 Conclusion

This study provides one of the most comprehensive assessments of district-level fertility in Botswana to date. By combining direct census estimation with comparative model-based techniques, it demonstrates the reliability of census data while capturing the heterogeneity of fertility across the country. The results reveal a Botswana firmly in transition: urban districts approaching or falling below replacement fertility, rural districts sustaining high fertility linked to mortality and socioeconomic disadvantage, and transitional areas reflecting mixed trajectories.

The implications are clear: fertility decline in Botswana is uneven, shaped by structural inequalities in education, employment, and health. Policies must therefore adopt a differentiated approach, targeting rural areas with interventions to reduce mortality and expand women's opportunities, while preparing urban areas for the socioeconomic consequences of very low fertility. Future research should integrate longitudinal and qualitative perspectives to better understand the behavioural drivers of fertility decisions at district level. Ultimately, the study highlights that achieving equitable fertility transitions across Botswana will require not only demographic monitoring but also sustained social investment that addresses the root determinants of reproductive behaviour.

5.0 References

1. Aitken, R. J. (2024). What is driving the global decline of human fertility? Need for a multidisciplinary approach to the underlying mechanisms. *Front Reprod Health, 6*(1364352). doi:10.3389/frph.2024.1364352

2. Statistics Botswana. (2025). *Botswana's Population and Hosuing Census 2022: Analytical Report Volume 1.* Gaborone: Statistics Botswana

3. Statistics Botswana. (2025). *Botswana's Population and Housing Census 2022: Analytical Report Volume 5.* Gaborone: Statistics Botswana.

4. Moultrie, T. A., Dorrington, R. E., Hill, A. G., Hill, K., Timæus , I. M., & Zaba, B. (2013). *Tools for Demographic Estimation.* Paris: International Union for the Scientific Study of Population. Retrieved from demographicestimation.iussp.org

5. Abate, B. B., Sendekie, A. K., Ayele, M., Lake, E. S., Wodaynew, T., & Tilahun, B. D. (2024). Mapping fertility rates at national, sub-national, and local levels in Ethiopia between 2000 and 2019. *Front. Public Health, 12*(1363284). doi:10.3389/fpubh.2024.1363284

6. Bainame, K., & Letamo , G. (2014). Fertility Levels and Trends in Botswana. *Botswana Notes and Records, 46,* 37-45.

7. Behrman, J., & Gonalons-Pons, P. (2020). Women's employment and fertility in a global perspective (1960-2015). *Demogr Res, 43,* 707-744. doi:10.4054/demres.2020.43.25

8. Nestorová Dická, J., & Lipták, F. (2024). Regional fertility predictors based on socioeconomic determinants in Slovakia. *J Pop Research, 40*(20). doi:10.1007/s12546-024-09340-3

9. Statistics Botswana. (2022). *2022 Population and Housing Census Preliminary Results V2.* Gaborone: Statistics Botswana.

10. Statistics Botswana. (2025). *Population and Housing Census 2022: Adminitrative and Technical Report V2.* Gaborone: Statistics Botswana.

11. Pebley, A. R., Casterline, J. B., & Trussell, J. (1982). Age at First Birth in 19 Countries. *International Family Planning Perspectives, 8*(1), 2-7. doi:10.2307/2947812

12. American Society for Reproductive Medicine. (2012). *Age and Fertility: A Guide for Patients.* Washington, D.C: American Society for Reproductive Medicine.

13. Rele, J. R. (1967). *Fertility Analysis Through Extension of Stable Population Concepts.* Berkeley: Institute of International Studies University of California.

14. Hauer, M., Baker, J., & Brown, W. (2013). Indirect Estimates of Total Fertility Rate Using ChildWoman/Ratio: A Comparison with the Bogue-PalmoreMethod. *PloS ONE, 8*(6).

15. Ponnapalli, K. M., & Soren , R. K. (2018). Indirect Estimation of Selected Measures of Fertility and Marital Fertility from information on CWR (0-9):An application of India, States/Districts. *Momona Ethiopian Journal of Science, 10*(1), 89-108.

16. Hauer, M., & Schmertmann, C. (2020). Population Pyramids Yield Accurate Estimates of Total Fertility Rates. *Demography, 57,* 221-241. doi:doi.org/10.1007/s13524-019-00842-x

17. Schober, P., Boer, C., & Schwarte, L. (2018). Correlation Coefficients: Appropriate Use and Interpretation. *Anesthesia & Analgesia, 126*(5), 1763-1768. doi:10.1213/ANE.0000000000002864

18. Crombach , L. G., & Smits , J. (2024). Understanding the urban-rural fertility divide in sub-Saharan Africa: The critical role of social isolation. *Population, Space and Place, 30*(8), e2801. doi:10.1002/psp.2801

19. Tesfa, D., Tiruneh, S. A., Gebremariam, A. D., Azanaw, M. M., Engidaw, M. T., Kefale, B., & Abebe, B. (2023). The pooled estimate of the total fertility rate in sub-

Saharan Africa using recent (2010–2018) Demographic and Health Survey data. *Front Public Health, 10*(1053302). doi:10.3389/fpubh.2022.1053302

20. Biney, E., Amoateng, A., & Ewemooje, O. (2020). Patterns of fertility in contemporary South Africa: Prevalence and associated factors. *Cogent Social Sciences, 7*(1). doi:10.1080/23311886.2020.1858575

21. Mugisho, G. (2025). How conflict shapes the impact of infant and child mortality on fertility in the Democratic Republic of the Congo. *Advances in Life Course Research, 65*(100692). doi:10.1016/j.alcr.2025.100692

Funding Declaration

The study did not receive any funding.
