# Supplementary material for: Estimation of district-level fertility using age-structured census data and assessment of spatial–socioeconomic differentials in Botswana, 2022
Source: PLoS One. 2026 Feb 5;21(2):e0342090. doi: 10.1371/journal.pone.0342090 (PMC12875440; doi:10.1371/journal.pone.0342090)
Supplement: Attachment — Submitted filename: Response to Reviewers comments.docx [file pone.0342090.s002.docx]

**Estimation of District-Level Fertility using Age-Structured Census Data and Assessment of Spatial–Socioeconomic Differentials in Botswana, 2022**

**Response to Reviewer #1**

We sincerely thank Reviewer #1 for the careful and constructive evaluation of our manuscript. The comments were instrumental in improving the clarity, structure, and scientific rigour of the paper. Below, we provide a detailed response to each comment and describe the specific revisions made in the amended manuscript.

| **REVIEWER’S COMMENTS** | **RESEARCHER’S RESPONSE** |
| --- | --- |
| 1. ABSRACT   The abstract is missing a clear presentation of standard demographic procedures, key findings, and comparisons between direct estimates and model-based fertility measures and summarized conclusions. The abstract need to reflect the analytical scope of the paper. | We fully agree with this assessment. The abstract has been substantially revised to clearly reflect the analytical scope of the paper. Specifically:   - **Standard demographic procedures** are now explicitly stated, including the calculation of ASFRs and TFRs from census data. - **Comparative validation** against established indirect and model-based methods (Rele; Hauer et al.; Ponnapalli & Soren; Hauer & Schmertmann) is clearly articulated. - **Key empirical findings** are summarised, including national-level convergence (TFR 2.6–3.0) and pronounced district-level heterogeneity. - **Conclusions and policy implications** are now explicitly stated.   These revisions ensure that the abstract now provides a complete, self-contained summary of methods, findings, and implications. |
| 2. **Introduction**   The demographic context in Botswana should, which is explicitly highlighted in the methods, may be could be more communicative in the introduction part. | We have strengthened the demographic context in the Introduction by:   - Expanding discussion of Botswana’s long-term fertility transition using historical evidence (Bainame & Letamo). - Explicitly situating district-level heterogeneity within broader processes of urbanisation, education expansion, and labour force participation. - Clarifying why national averages obscure meaningful subnational fertility dynamics.   This ensures that the demographic motivation for the study is clearly established before the Methods section. |
| The paragraph beginning with “The 2022 PHC introduced major methodological innovations…” should also be moved to the Introduction. | This paragraph has been **relocated from the Methods to the Introduction,** where it now provides contextual background on data quality, digital enumeration, and census reliability. The Methods section is now strictly confined to analytical procedures. |
| The paragraph beginning with “The contribution of this study is both methodological and substantive…” is appropriate as the final paragraph of the Introduction. | We agree and have retained this paragraph as the **final paragraph of the Introduction,** unchanged in substance but now better positioned following the expanded contextual discussion. |
| A reference to WHO should be added where socioeconomic determinants are defined or classified. | A reference to WHO frameworks has now been incorporated in the Introduction where socioeconomic determinants (education, employment, marital patterns, and access to services) are conceptually defined. This strengthens the theoretical grounding and aligns the study with established public health classifications. |
| 3. **Methodology**   Information that presents results, outputs, or interpretation should not appear in the Methods section. | We have carefully reviewed the Methods section and **removed all interpretive or results-oriented statements.** The section now strictly describes:   - Data sources - Quality assessment procedures - Estimation techniques - Validation strategy - Measurement of socioeconomic and mortality indicators   All interpretation and trend discussion has been relocated to the Findings or Discussion sections. |
| Mitigation measures to quality issues highlighted in the paper should come out more specific and explicit. | This issue has been addressed by expanding **Section 2.2 (Data Quality Assessment and Mitigation Measures).** The revised section now explicitly details:   - Parity and recent-birth consistency checks - Aggregation strategies for small districts - Triangulation across multiple indirect methods - Criteria used to prioritise estimates where discrepancies arise - Cautious interpretation in migration-affected districts   These additions clarify how potential biases were actively managed rather than merely acknowledged. |
| 4. **Findings**   Only empirical results should appear in the Findings section. | We have carefully revised the Findings section to ensure that it now reports **only empirical results**. Specifically:   - Interpretive comparisons (e.g., “Similar patterns are evident in Francistown…”) have been moved to the **Discussion.** - Normative or evaluative statements have been removed from the Findings. |
| Statements linking socioeconomic factors and fertility require statistical justification. | We agree and have strengthened this section by:   - Explicitly grounding statements in **observed district-level associations** presented in Table 3. - Clarifying that relationships are **descriptive and correlational**, consistent with census-based cross-sectional data. - Reserving causal interpretation for the Discussion, where findings are situated within demographic transition theory and comparative literature. |
| Some findings currently read as conclusions. | Statements such as “These findings reinforce the utility of combining methods…” have been **relocated to the Discussion and Conclusion,** ensuring that the Findings section remains strictly empirical. |
| Table 2 does not clearly demonstrate the proposed relationship between age distribution and TFR. | Table 2 has been **restructured**, and its interpretation clarified in the text to explicitly link age distribution patterns with observed TFR variation across districts. The discussion now focuses on percentage distributions and demographic composition rather than implicit inference. |
| The manuscript repeatedly restates the objectives. | We have streamlined the manuscript by:   - Stating the objectives **once clearly in the Introduction** - Referring back to them implicitly rather than restating them verbatim in later sections   This has reduced redundancy and improved narrative flow. |
| **6. Summary and Conclusion**  The summary and conclusion should be reinforced with clearer statements on variation significance and direct vs. model-based differences. | The Discussion and Conclusion have been strengthened to:   - Clearly articulate the **significance of district-level variation** - Explicitly discuss **where and why direct and model-based estimates diverge** - Highlight implications for **policy, planning, and demographic monitoring** |
| **7. Overall Assessment**  Strengthening analytical presentation and repositioning content will enhance coherence and credibility. | We appreciate this summary and believe the revisions directly address these concerns. The manuscript now demonstrates:   - Clear separation of Methods, Findings, and Discussion - Stronger statistical and demographic justification - Improved coherence and scientific credibility   We are grateful for the reviewer’s insightful guidance, which has substantially improved the manuscript. |
